# The role of government in public health and public health crises: A perspective from Alberta

**DOI:** 10.1177/08404704251359463

**Published:** 2025-09-18

**Authors:** Braden J. Manns, Stephanie E. Hastings

**Affiliations:** 1 2129 University of Calgary, Calgary, Alberta, Canada

## Abstract

Public health leaders and programs have played an important role in Alberta’s response to major health events, including COVID-19. We discuss how the Alberta provincial government has exerted increasing control over public health functions in the province, and how this played out over the course of the pandemic and beyond. Given the significant role that the social determinants of health play in shaping the health of the population, we also discuss how successive governments have approached policy and investment in public health. Using our experiences, we discuss a more effective role for government in a public health crisis, and in the health of the public more broadly. We advocate for a Health in All Policies approach to government decision-making and a move towards focusing on evidence rather than ideology.

## Introduction

Many Canadians probably could not have defined public health prior to 2020, let alone named their Chief Medical Officer of Health (CMOH). But a few months into the COVID-19 pandemic, CMOHs were widely regarded as heroes. People wore t-shirts emblazoned with their names and faces, the outfits they wore for nightly news updates inspired copycats, and families discussed them around the dinner table.^1,2^

The COVID-19 pandemic reminded all of us about the importance of public health and is a useful starting point for this article before we discuss the role of government in public health more broadly. The direct impact of the pandemic was huge—over seven million lives lost worldwide. Disability from COVID-19 has changed many forever.

Public health restrictions were needed to prevent many more deaths and to prevent health systems from being overwhelmed. And they made a difference. Jurisdictions with tighter public health rules had lower COVID-19 death rates, though there were differences in the effectiveness of different measures.^3-5^ Canada had lower death rates than most other comparator countries, in part due to our use of public health measures.^6^

But there were side effects. The pandemic—and measures restricting the public’s activities—caused massive economic hardship, especially for society’s most vulnerable. It caused more social isolation, leading to more substance use and mental health disorders. It stretched healthcare workers and systems to—or past—the breaking point. Children, especially our most vulnerable ones, were left with educational lags.

As the pandemic’s waves dragged on, public health measures became increasingly polarizing, creating deep rifts in society. Clear conflicts emerged between Alberta’s provincial government, the CMOH in whom they had initially placed so much trust, the province’s healthcare system, and some groups in society.

The pandemic exposed longstanding tensions between politicians, who seek re-election every 4 years and often focus on short-term wins, and public health teams who rely on evidence to advocate for better population health, often requiring long-term planning beyond election cycles. This misalignment, including tensions between ideology and evidence, is particularly evident in Alberta right now. This theme will weave throughout this article.

In March 2020, our regular jobs with Alberta Health Services (AHS) as Associate Chief Medical Officer (BJM) and Acting Director of Health Systems Evaluation and Evidence (SEH) were largely put on hold. We were tasked, along with others, with establishing the COVID-19 Scientific Advisory Group. Reporting directly to the groups making the relevant decisions, including the AHS Executive Leadership Team, the AHS Emergency Coordination Centre which coordinated the pandemic health system response, and Alberta’s CMOH, the Scientific Advisory Group summarized evidence on a wide range of topics and made evidence-based recommendations.

Subsequently, BJM spent 5 months as the Interim Vice President and Medical Director of Clinical Operations for AHS during the Delta wave of the pandemic, when Intensive Care Units (ICUs) were nearly overwhelmed. In June 2022, BJM was asked to cover another Interim VP role, this time for provincial clinical programs, including AHS public health. Both authors took other roles at the University of Calgary in June 2023. What follows are our own opinions about public health in Alberta. We acknowledge that our perspective is just one of many and that Indigenous leaders, community organizations, and other groups may see things differently.

## The structure of public health in Alberta

**Figure 1.**
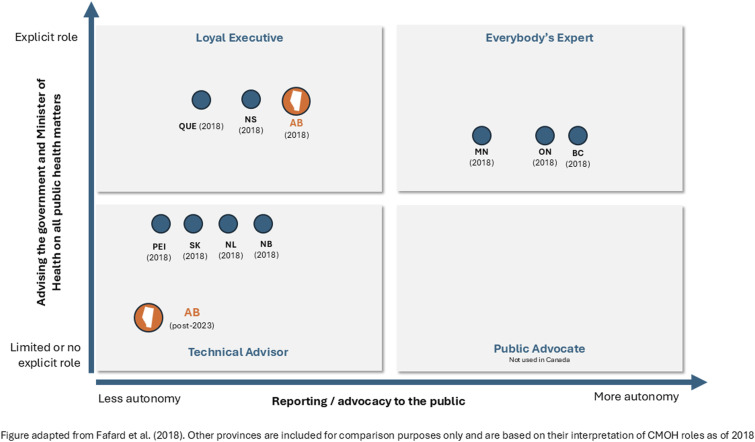
CMOH models in Canada.

Each province is responsible for organizing healthcare, resulting in wide variability in how health systems—including public health—are set up.^7^ In a 2018 study, Fafard et al. identified three CMOH models in Canada.^8^ In each, CMOHs attempt to protect public health, but the models vary mainly based on two dimensions: the extent to which they advise the Health Minister and government, and to which they have an independent advocacy role with the public (Figure 1). The “Loyal Executive” mostly supports and advises the government, as well as designs and delivers public health functions. “Everyone’s Expert” has an advisory role but more independent authority to communicate with the public. Finally, the “Technical Officer” has no legislated advisory or advocacy roles, though in practice provides some advice to government and may communicate to the public in emergencies.

In Alberta, the Ministry of Health sets the policy, legislation, and standards for the health system.^9^ At the start of the pandemic, the Loyal Executive-type CMOH^8,10^ provided recommendations to the minister and directives to the health authority (Alberta Health Services or AHS) on matters of public health.

The provincial health ministry, through the CMOH, provided guidance and directives to AHS for the management of all notifiable diseases, which were then operationalized by AHS across its facilities. AHS medical officers of health, through their oversight of various public health, surveillance, and disease control teams, also used these directives within the community.

## Alberta’s increasing political influence over public health during the pandemic

The functions outlined above were clear early in the pandemic. The government relied on the CMOH for scientific expertise and acted on her evidence-based guidance, letting her be the “face” of the pandemic response in many cases. AHS, the provincial health authority, implemented policies based on the CMOH’s direction.

**Figure 2.**
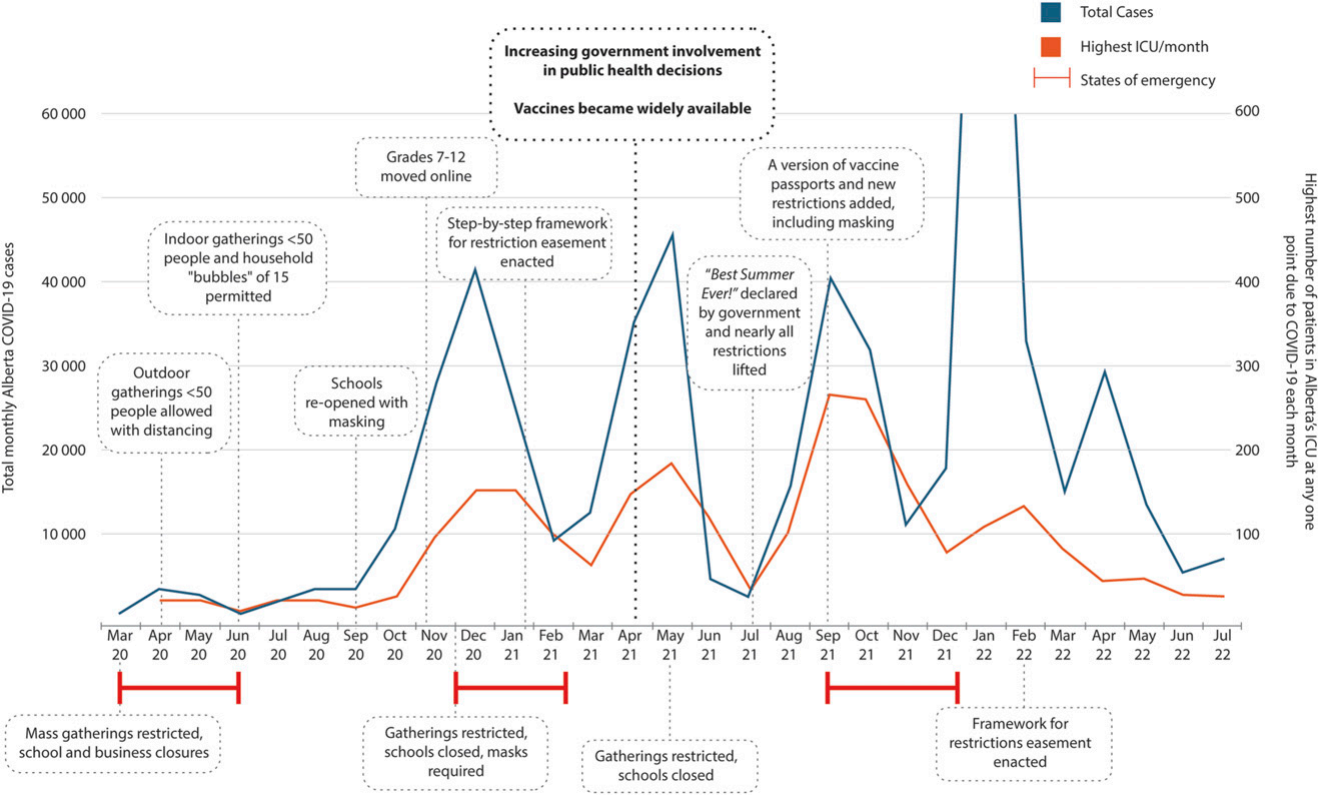
Alberta’s major COVID-19 public health measures, cases, and ICU patients, March 2020-July 2022.

This changed over time, and it became clear that the CMOH was simply providing advice to Cabinet, the actual decision-maker for public health rules. Until 2023, the provincial Public Health Act required that in public health emergencies, decisions come from the CMOH. But testimony in a lawsuit filed against the province’s public health restrictions confirmed that decisions were actually being made by the government’s own COVID cabinet committee,^11^ with declining influence by the CMOH over time. Figure 2 shows a timeline of major COVID-19 public health decisions and their relationship to COVID-19 cases and ICU hospitalizations in Alberta.

Given government’s concerns around who should make decisions, Premier Smith appointed Preston Manning to review legislation and governance practices used in Alberta’s public health emergencies.^12^ It became clear though that his report had largely been written and its conclusions made public *before* it was even commissioned by the Premier.^13^ Not surprisingly, the Alberta government acted on those recommendations, amending the Public Health Act in 2023 to affirm that elected officials have final decision-making authority,^14^ aligning with how public health decisions were actually made in the later stages of the pandemic, and moving the CMOH to the “Technical Officer” model (Figure 1).

The Manning report also recommended that “evidence-informed decision-making consider non-scientific evidence” and that “alternative scientific narratives and hypotheses” should be given weight in future public health emergencies.^12^ Although the government has yet to address whether or how this might be enacted going forward, the Premier also appointed a taskforce, a small group staunchly opposed to vaccines and public health measures, to review Alberta’s COVID-19 response.^15^ The report covers a range of topics and is filled with conspiracy theories and anti-science sentiment, including false assertions that vaccines caused thousands of deaths. The taskforce recommended immediately halting use of all COVID-19 vaccines and recommended using medications, including ivermectin, that have been rejected by mainstream science after extensive study. The report outraged Alberta’s mainstream medical community^16^ and the Canadian Medical Association issued a statement expressing their alarm.^17^ Nonetheless, in June 2025, the government announced it would no longer publicly fund COVID-19 vaccines for most people.^18^ Albertans—including most seniors—will now need to pay out of pocket to be protected from the virus.

After firing its CMOH in 2022, the Government of Alberta appointed a seasoned health system leader as Interim CMOH. He left that role in 2025, in part because he was not allowed by government to communicate with the public during a large Measles outbreak.^19,20^

In May 2025, the government announced major changes to the structure of public health in Alberta. While the details remain unclear, some frontline public health functions are moving to a new provincial primary care organization, creating an opportunity for greater synergy between primary care and public health. Other roles, including medical officers of health, public health inspectors, and policy staff will move into the Ministry.^21^

Since 2022 in Alberta, we have observed blatant disinformation in government reports on public health,^12,15^ an undermining of the CMOH role, and now a tightening of control over key public health functions within government. This has created major concerns for medical officers of health, and other public health staff, and should raise alarms with Albertans. It risks further eroding public trust not just in the Alberta government, but also in public health professionals and mainstream science.^22,23^ It has raised legitimate concerns about whether the government will follow scientific evidence, putting Albertans’ health at risk, including in future public health emergencies.

While the changes in Alberta were the most extreme example among provinces, they echo trends across Canada discussed by McLaren et al.^24^: a downgrading of the status of public health activities within health authorities, loss of independence of CMOHs, decreasing funding, and a reduction in the scope of public health.

## What’s the role of government in the health of the public more broadly?

**Figure 3.**
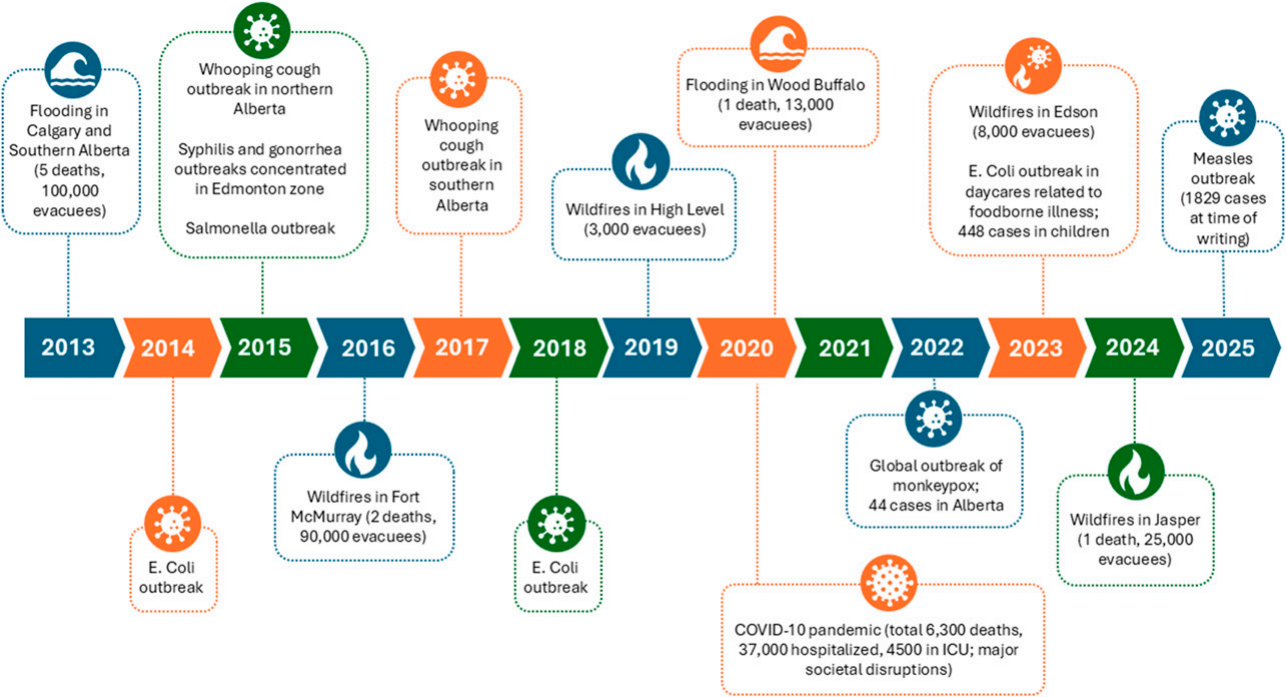
A non-exhaustive overview of Alberta’s major public health events since 2013.

COVID-19 is far from the only public health event Alberta has faced in recent years. Figure 3 illustrates a timeline of recent Alberta events, highlighting the importance of having high-functioning public health teams to rapidly respond in times of need. Public health teams are also critical for floods and fires, caring for those evacuated, reducing injury, and providing support to prevent further infectious illness in crowded conditions.

But on a broader scale, governments (and public health) have other critical roles in shaping the health of their population outside of public health emergencies. After steady increases over the last century, life expectancy at birth for Canadians has declined since 2019,^25,26^ only partly due to the pandemic. Many factors outside healthcare influence health, including economic and social policies and our political systems.

Alberta’s political reliance on ideology over evidence is apparent in other public health areas too. For example, the government has recently introduced the Compassionate Intervention Act, which will allow people with substance addiction issues to be forced into treatment. It has committed $180 million towards building “intervention centres” where those found eligible will be unable to refuse medications, observation, and treatment advice.^27^ Without question, Albertans are concerned with the increasing number of drug-related deaths, including among the unhoused. People are keen to see action, but there is no good evidence that supports involuntary over voluntary treatment, and there are serious concerns about breaches of patients’ civil liberty.^28^

On the other hand, there *is* high-quality research showing how to prevent people from entering addiction in the first place, and improve population health overall, suggesting an alternate place for government investment.

Researchers compared health outcomes in over 3,000 counties across 45 states in the United States,^29^ examining the impact of modifiable determinants of health including:

• social and economic factors (e.g., community safety, education, employment, family and social support, and income);
• physical environment (e.g., air and water quality, housing, and transit);
• health behaviours (e.g., alcohol use, diet, exercise, and tobacco use); and
• healthcare (e.g., access to, and quality of, healthcare).

By studying how much each of these factors explained differences in how long people lived and their quality of life across these counties, researchers were able to explain over half of the variation in health outcomes. The rest of the variation was due to factors the study couldn’t measure, like genetics. Of the explained variation, nearly half (47%) of health outcomes were explained by social and economic factors, 3% by the physical environment, 34% by health behaviours (which are largely determined by social and economic factors and the physical environment) and only 16% by healthcare.

This isn’t a new finding, and the results are consistent with many other similar studies, which also show that modifiable determinants of health are highly correlated with future substance use.^30^ It’s clear that governments can influence health through their economic and social policies, spending on social programs and education, and public health divisions staffed by experts in how those policies and interventions impact the health of the population.

Despite this, Canada’s spending on social programs continues to decline relative to expenditures on healthcare. Fifty years ago, Canada’s largest provinces spent 16%-36% *more* on social and education programs than on healthcare. Provinces now spend 5%-28% *less* on social and education programs than they do on healthcare. Compared to many European countries, Canada spends about half as much on social programs—like employment insurance, old age security, disability, family support programs, housing supports, and the like. The same is true in Alberta.^24^

To summarize, governments spend much more on treating illness than preventing it.^31^ Although it’s clear that investments in policies and interventions that mitigate the social determinants of health can improve people’s health, the benefits are only visible years down the road. And the investments aim to reduce inequality, meaning more for the poor—not always popular with voters who might not benefit directly. Instead, in the face of long waits in the healthcare system, politicians favour investments in healthcare that may lead to changes within the 4-year electoral cycle.

## Recommendations for the government in a public health emergency

Whatever the emergency, protecting lives requires action from all levels of government, public health, and most importantly, the public. Collaboration is critical. So too is ensuring public trust in government, which can be fostered by adhering to best evidence and transparent communication.

The evidence on how best to manage all aspects of a public health emergency should be efficiently reviewed and summarized by independent national groups, with coordination by the Public Health Agency of Canada. The National Advisory Committee on Immunization does this for vaccinations, but we also need evidence for public health measures, mental health supports, and many other topics. Such evidence is critical to design better public health policy, and must be available quickly. Since the best evidence doesn’t differ in Newfoundland or British Columbia, having a federal body bring together high-quality syntheses would support consistent and concerted responses across Canada.

While some provinces, including Alberta and Ontario, established their own advisory committees during the pandemic, reviewing evidence in each province is time-consuming and duplicative. Our experience with Alberta’s Scientific Advisory Group suggests there could be a role for provincial committees that use evidence syntheses created nationally; add in local data and perspectives, including the impact of policies on marginalized groups; and make evidence-based recommendations to guide decision-making across a broad range of issues of relevance to public health.

Governments, public health, and health systems must communicate transparently with the public they serve, acknowledging that information may change as the emergency goes on. They should weigh the impact of their decisions, particularly around public health rules, recognizing that these decisions will never be popular with all groups and will always have unintended impacts. Acknowledging that the effectiveness of different public health measures—particularly as it relates to infectious spread—varies,^3-5^ governments must choose the most effective measures that minimize the impact on the economy, on vulnerable populations, and on our children.

Governing during a pandemic is hard, but maintaining focus on best evidence and transparent communication will help everyone understand why decisions are being made and minimize unintended harms. During public health events, governments must let the experts, including the CMOH, speak.

## Recommendations for the government for public health more broadly

Governments must acknowledge and accept the importance of public health and that they have a critical role in the health of their population. They must remember that investments in education and social programs influence health, and that investments in healthcare mean less money for programs that mitigate the social determinants of health. Governments must better balance their investments across healthcare and social programs.

It’s not just about investment. It’s also about ensuring that broader legislation doesn’t adversely impact population health. Governments at all levels are constantly introducing new legislation, taxes, and policies that have effects beyond the area they are intending to impact. That’s led to calls for a Health in All Policies approach, where the health of the population is explicitly considered by all levels of government in their decision-making^32^—even for decisions that seemingly aren’t related to health. The goal of this type of approach is to reduce the impact of social determinants of health, promote health, and prevent disease.

Finland has been a leader in a health-centric approach since the 1970s, focusing on how policies or taxes would impact the development of chronic diseases like heart disease and stroke, by impacting the risk factors that lead to these conditions (i.e., poor diet, obesity, smoking, and exercise). The Finnish government formally incorporated Health in All Policies in 2000—justified given the striking improvements in healthy diet and drastic reduction in deaths due to heart disease, which dropped to around 20% of what they were in the 1970s.

To date, Quebec and Newfoundland are the only provinces in Canada to formally adopt many of the elements of a Health in All Policies approach. While capacity building and skills would clearly be required, this would be a solid investment for Alberta and other provinces.

## Recommendations for health leaders

Policy direction for public health comes from government, not health systems. But health systems still have an important role in population health. With regionalization, health leaders have some flexibility in how budgets are spent.^7^ They can allocate more of their budgets towards prevention and equity in the spirit of achieving the quintuple aim—acknowledging this will compete with hospital budgets. They can also create better integration between the health system (primary care and health authorities), community providers, and the social care system, ensuring patients impacted by the social determinants of health get the supports they need to stay healthy, and out of hospital.

Governments have a critical role in public health, during public health events, but perhaps more importantly, outside such events. Indeed, we would argue their most important role is shaping the health of our population—including through creating a strong sustainable economy, evidence-based legislation, and its focus on education, public safety, and the environment. It all impacts our health.
